# MCMNET: Multi-Scale Context Modeling Network for Temporal Action Detection

**DOI:** 10.3390/s23177563

**Published:** 2023-08-31

**Authors:** Haiping Zhang, Fuxing Zhou, Conghao Ma, Dongjing Wang, Wanjun Zhang

**Affiliations:** 1 School of Computer Science, Hangzhou Dianzi University, Hangzhou 310018, China; 2 School of Information Engineering, Hangzhou Dianzi University, Hangzhou 310005, China; 3 School of Electronics and Information, Hangzhou Dianzi University, Hangzhou 310018, China; shiyichen1213@gmail.com (F.Z.);

**Keywords:** action detection, multi-scale, self-attention mechanism

## Abstract

Temporal action detection is a very important and challenging task in the field of video understanding, especially for datasets with significant differences in action duration. The temporal relationships between the action instances contained in these datasets are very complex. For such videos, it is necessary to capture information with a richer temporal distribution as much as possible. In this paper, we propose a dual-stream model that can model contextual information at multiple temporal scales. First, the input video is divided into two resolution streams, followed by a Multi-Resolution Context Aggregation module to capture multi-scale temporal information. Additionally, an Information Enhancement module is added after the high-resolution input stream to model both long-range and short-range contexts. Finally, the outputs of the two modules are merged to obtain features with rich temporal information for action localization and classification. We conducted experiments on three datasets to evaluate the proposed approach. On ActivityNet-v1.3, an average mAP (mean Average Precision) of 32.83% was obtained. On Charades, the best performance was obtained, with an average mAP of 27.3%. On TSU (Toyota Smarthome Untrimmed), an average mAP of 33.1% was achieved.

## 1. Introduction

Due to the rapid growth of online video platforms, video understanding has attracted the attention of a large number of researchers in recent years. Action recognition and action detection are two fundamental tasks in the field of video understanding. Action recognition is the classification of action instances within a pre-edited video. In terms of temporal action detection, it involves the temporal localization and classification of multiple action instances within a raw, unedited video, requiring the detection of the start and end times of a specific action instance and its classification. Compared with action recognition, temporal action localization is closer to realistic scenarios. Therefore, temporal action detection is more challenging than action recognition while also being closer to practical applications. Temporal action detection can be used for video retrieval to filter out some videos containing inappropriate content in a large amount of video data. It can also be applied to security, where violent behavior can be detected by cameras.

**Figure 1:**
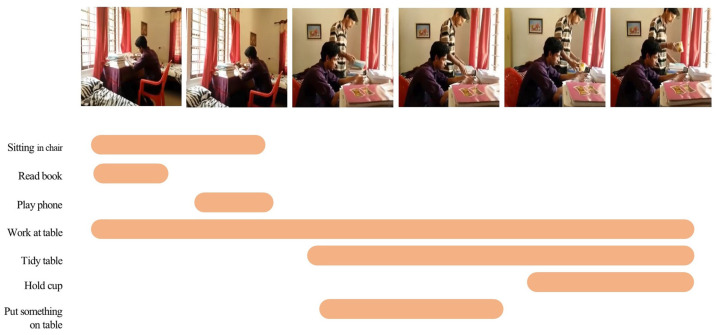
Complex temporal relations in daily life videos. Here, we show a common distribution of action duration in a daily life video, which includes both long-range and short-range dependencies among actions.

Especially for videos that contain a large number of action instances, the duration of these actions can vary, and there may be overlapping parts between them. Such videos often better reflect our daily lives. For example, a video of a person working at a table (see Figure 1) may contain multiple different action instances, such as sitting in a chair, reading a book, tidying a table, holding a cup, etc. The duration of these actions vary significantly, and the temporal relationships between actions are also very complex, such as a person holding a cup while tidying a table.

Towards modeling such complex temporal information, previous approaches have partly used convolutional networks [1,2,3,4,5]. These methods perform well at aggregating short-term temporal information. However, they are limited by the size of the receptive field of the convolutional network, which prevents them from capturing the relationships between distant segments in the video. Some researchers subsequently found that constructing a graph structure using each video frame as the node and the temporal relationship between video frames as the edge of the graph convolution [6,7,8,9] could well model the temporal information between video frames. However, the performance of these methods depends on how the graph structure is constructed and the choice of some hyperparameters. With the advent of vision Transformers (ViTs) [10], transformer-based methods [11,12,13,14,15] quickly emerged. The ability of the self-attention mechanism to capture long-term dependencies allows it to model the global temporal context better. However, a pure transformer network requires much more memory than a convolutional network when fed with a large amount of data simultaneously. It is also essential to take into account the local temporal context information.

**Figure 2:**
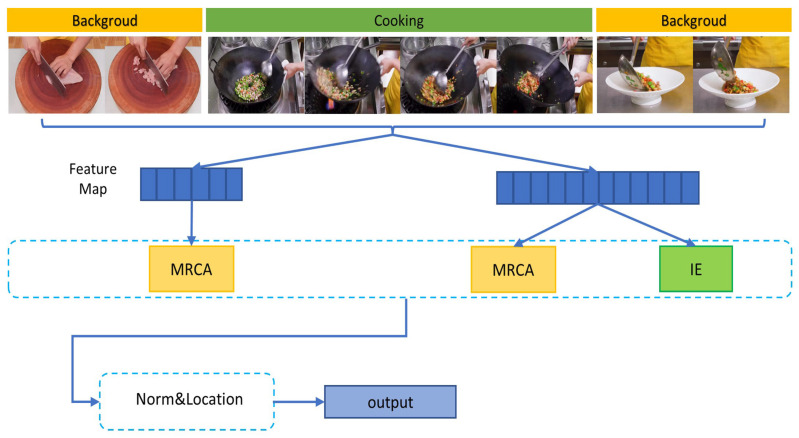
Overview of our proposed approach. MCMNET uses MRCA to construct video features with multi-scale temporal information. The IE module, as supplementary to MRCA, uses dilated convolution to capture long-range and short-range temporal context, which makes the video feature ampler. Norm&Location regularizes the data and produces the result.

Therefore, in order to model contextual information at different temporal scales more effectively, we designed a two-stream network MCMNET, as shown in Figure 2, by first splitting the input data into two temporal resolution streams, with the aim of obtaining a richer representation of information with different coarse and fine granularity by processing more raw feature information with different temporal resolutions. Both streams are fed into the Multi-Resolution Context Aggregation module (MRCA) to obtain multi-scale temporal features. The MRCA module is composed of Attenuation blocks and Aggregation blocks. The Attenuation block mainly consists of a Reduction block, Global block, and Local block which aim to build a pyramid of features with different temporal resolutions. The Reduction block operates on the temporal resolution, reducing the resolution and increasing the dimension of the features; the Global block uses multi-headed self-attention mechanism to model global temporal features; and the Local block uses multiple convolutional layers to model local information. Followed by the modeling of temporal relations at different scales, an Aggregation block is used to fuse the features from each stage to get a unified feature representation. In order to aggregate long-range and short-range contexts more efficiently, we have added an Information Enhancement (IE) module to the back of the high-resolution stream as a complement to the MRCA module. The IE module is a stack of Multi-Path Temporal Convolutions. In each convolution block, there are three paths: the long-range path, to expand the perceptive field and aggregate the long-range context by dilated convolution; the short-range path, to aggregate the short-range context by the ordinary convolution; and the original path, to enhance the representation of features and to solve the problem of network degradation during training. Finally, we combine the three paths and perform element-wise addition to obtain a strong feature with long-range and short-range contexts. We summarize our contributions as follows.

(1) We propose an effective two-stream network to aggregate the multi-scale temporal context. Using this model, we are able to detect action in some scenarios where the temporal relation of the action is complex.
(2) A multi-scale context modeling network is proposed for temporal action detection. MCMNET consists of two main modules: MRCA and IE. MRCA processes the input data in multiple stages with different temporal scales, which allows MCMNET to learn both fine-grained relations in the early stage and coarse relations between composite actions in the latter stage. While IE is used to aggregate long-term and short-term context effectively, which makes the video features richer.
(3) The experiments prove the convincing performance of MCMNET on three popular action detection benchmarks: ActivityNet-v1.3, Charades, and TSU.

## 2. Related Work

In this section, we review the prior work related to action recognition, action detection with CNN, and action detection with transformer.

### 2.1. Action Recognition

Action recognition is an important task in video understanding. Most of the traditional methods were based on hand-designed visual features [16]. Later on, with the success of deep learning, most of the methods are now based on neural networks. From the beginning, there were dual-stream networks [17,18,19], which used both optical and RGB streams as input and sent to a 2D convolutional neural network for processing. This was followed by the 3D convolutional network [20,21,22], which uses a 3D tensor with two spatial and one time dimension to model spatio-temporal features. To reduce the computational consumption of 3D convolutional networks, some approaches split the 3D convolution into 2D convolution and 1D convolution, becoming (2+1)D convolution [23,24,25]. We are inspired by the dual-stream network and set up a dual-resolution stream input in our network.

### 2.2. Action Detection with CNN

Action detection aims at localizing the temporal boundaries of human activities in untrimmed videos and classifying the action categories. Most existing work has used CNN-based models [4,26,27,28] to extract spatio-temporal features from the input video frames before feeding the features into the TAD (Temporal Action Detection) network. A common practice is first to generate temporal proposals and then classify each proposal to one of the action categories [3,29,30,31]. For generating proposals, there are anchor-based approaches [32,33,34], which retrieve fine-grained proposals by adjusting a pre-defined multi-scale anchor. There are also boundary-based approaches [35,36,37,38], which predict the start and end confidence of each frame and then match start and end frames to generate the proposals with confidence evaluation. Refs. [30,39] generated proposals based on pre-defined sliding window anchors and train a classifier to filter anchors. Another practice is the one-stage approach [40,41,42], which performs localization and classification at the same time, and thus, it is more efficient. Ref. [40] presented the first one-stage TAD method using convolutional networks. Later influenced by the anchor-free method [43,44,45] in the object detection task, AFSD, [46] designed a basic anchor-free localizer, along with making full use of the temporal insights of videos to propose novel refinement strategy and consistency learning. Moreover, [47] explored the combination of anchor-based and anchor-free methods. In our work, we define anchor points in the Norm&Location module and combine starting and ending predictions to make training more regular.

### 2.3. Action Detection with Transformer

The Transformer [48] approach was first applied to NLP, but later, with the advent of ViTs [10], the transformer was formally applied to the image domain. With the success of the transformer in the image domain, researchers began to use the transformer for various tasks in vision, including video understanding. ViViT [49], TimeSformer [50], and VidTr [51] propose to factorize along spatial and temporal dimensions on the granularity of encoder, attention block, or dot-product computation. Because transformer networks require large computational resources when processing larger data such as video, Video Swin Transformer [52] proposes shifted window attention to limit the computation within a small local window. While in the case of the TAD task. TadTR [53] is based on the structure of DETR and uses temporal deformable attention to solve the TAD task as a sequence prediction problem. Actionformer [54] uses an anchor-free approach to design a simple and pure transformer network. Our MRCA module inherits a transformer encoder architecture while gaining benefits from temporal convolution. This enables it to model global and local temporal context at different temporal scales.

## 3. Proposed Method

### 3.1. Problem Formulation

The input to our pipeline is a raw video that spans varying duration. Following common video action detection methods [55,56,57], we consider feature sequences extracted from video frames by a 3D CNN as input to MCMNET. For each video of length $l_{v}$, we divide it into T video clips; the length of each video clip is σ, $\left.T=l_{v}/\sigma \right.$, and the feature dimension corresponding to each video clip is C × 1. In this way, the input feature sequence for the pipeline can be written as $X={\left\{x_{i}\right\}}_{i=1}^{T}\epsilon R^{C\times T}$. Furthermore, for each video sequence, there is a set of labels with number N relative to it: $K={\left\{k_{n}=\left(t_{s,n},t_{e,n},C_{n}\right)\right\}}_{n=1}^{N}$, where $k_{n}$ represents the $n^{th}$ action instance, and $t_{s,n}$, $t_{e,n}$, and $C_{n}$ are its start time, end time, and action class, respectively. For each input video, temporal action detection model needs to predict M possible action instance $\Lambda ={\left\{\lambda_{m}=\left({\overset{-}{t}}_{s,m},{\overset{-}{t}}_{e,m},{\overset{-}{C}}_{m},P_{m}\right)\right\}}_{m=1}^{M}$. Here, $\lambda_{m}$ represents the $m^{th}$ predicted action in the video, it contains four indicators ${\bar{t}}_{s,m}$, ${\bar{t}}_{e,m}$, ${\bar{C}}_{m}$, and $P_{m}$. ${\bar{t}}_{s,m}$ and ${\bar{t}}_{e,m}$ represent the predicted start time and end time for the $m^{th}$ predicted action; ${\bar{C}}_{m}$ and $P_{m}$ are its predicted action class and confidence score, respectively.

### 3.2. MCMNET Architecture

**Figure 3:**
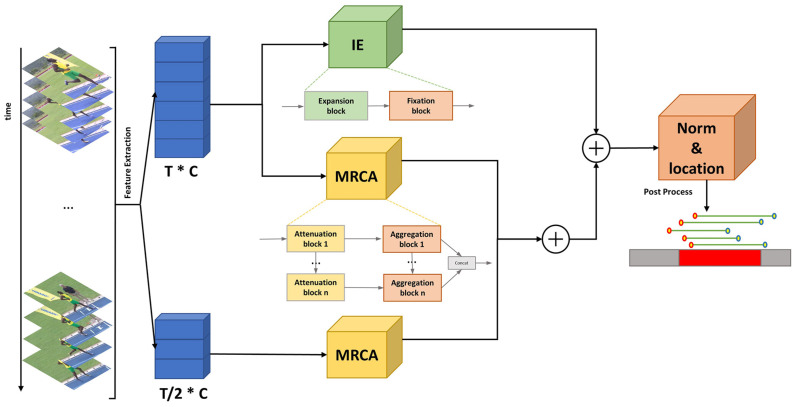
Overview of MCMNET architecture. The video is processed into two temporal resolution fragment characteristic sequences as input. MCMNET mainly includes three modules: MRCA, IE, and Norm&Location. First, MRCA is used to model multi-scale temporal context from two streams. At the same time, the high-resolution stream is input into IE for long-range and short-range timing coding to enrich feature information and enhance feature robustness. Then, the two stream features are fused into a stronger video feature. Finally, the feature is normalized through Norm&Location to produce results.

The overall architecture of MCMNET is illustrated in Figure 3. We pre-process the video to obtain two feature sequences with different temporal resolutions, which are used as input for the model. The model consists of three main modules: a multi-resolution context aggregation module (MRCA), an information enhancement module (IE), and a post-processing module (Norm&Location).

First, the fragment features are copied twice, and their temporal resolution is adjusted to T and T/2 by convolution, where the feature stream with a temporal resolution of T is called a high-resolution stream and another feature stream is called a low-resolution stream. The two streams will be passed through the MRCA module separately, and for the high-resolution stream, it will pass through the four stages of the MRCA. Each stage the dimensionality of the incoming data from the previous stage is changed, the temporal resolution will be reduced to half of the original one, and the channel size will be expanded to α times of the original one accordingly, where α is taken as 1.5 in the experiment. The dimensionality-changed data are then passed through a self-attention layer to obtain the global temporal context, after which the standard convolution is used to obtain the local temporal context. In this way, we try to have the model learn fine-grained action representation with more temporal information in the early stages and coarse-grained action reprsentation with less temporal information in the later stages. In order to increase the robustness and diversity of the information contained in the features, the same operation is applied to the low-resolution stream, with the difference that the temporal resolution decays at a rate of 2/3 and the channel size increases at the same multiplier.

Next, to improve the model’s ability to aggregate short-range and long-range contexts, we fed high-resolution stream into the IE module, which has eight blocks, each with an Expansion block and a Fixation block. There are three paths in the Expansion block, one of which uses dilated convolution to aggregate long-range context and expand the receptive field. One path uses regular convolution to aggregate short-range temporal context. The last path does not operate on the input features, keeping the original features for fusion with the other paths. However, the method of expanding the receptive field by stacking a large number of dilated convolution layers will cause gridding artifacts, which can lead to loss of information, so we effectively avoid this problem by adding a Fixation block after each Expansion block. There are also three path in the Fixation module, except that the dilated convolution in the long-range path is replaced by a convolution with a fixed dilated rate.

Finally, after fusing the features obtained above, they are fed into the Norm&Location module for regularization, after which the regression score and classification score are predicted by multiple fully connected layers and their losses are calculated separately, followed by back propagation.

### 3.3. Multi-Resolution Context Aggregation

**Figure 4:**
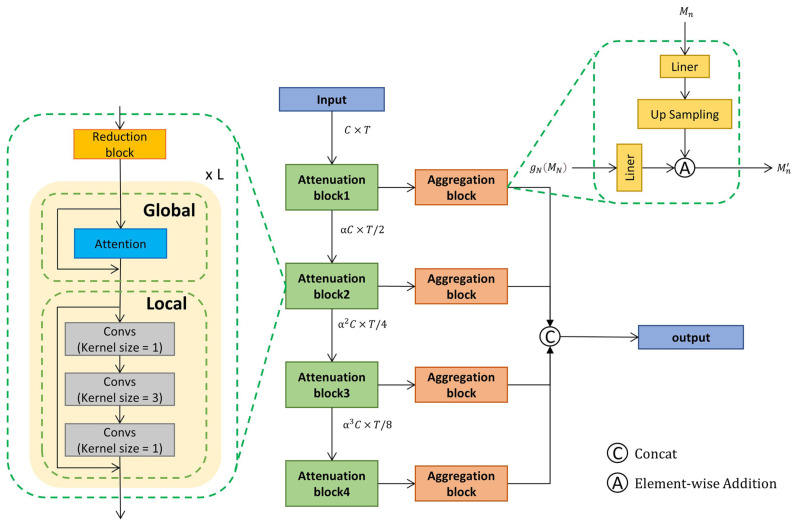
The detailed structure of the Multi-Resolution Context Aggregation module. There are four stages, each consisting of an Attenuation block and an Aggregation block, where the Attenuation block can be divided into: the Reduction block, which is used to change the temporal resolution while increasing the feature dimension; the Global block, which uses a self-attention mechanism to model global temporal information; and the Local block, which uses a convolutional network to model local contextual information. Furthermore, the Aggregation block uses a liner projection layer and upsampling to unify the video representation dimension.

The MRCA module is the core of modeling the temporal context, which processes the video sequence features obtained through the I3D network. As depicted in Figure 4, the MRCA contains four Attenuation blocks and four Aggregation blocks. Such multiple blocks are constructed to cope with the complex temporal relationships of the video while building a multi-scale hierarchy of temporal features.

**Attenuation Block.** The structure of the Attenuation block is shown in Figure 4, which can be subdivided into three main structures: the Reduction block, the Global block, and the Local block. In this stage, we use a temporal convolutional layer with kernel size and stride of 2 to decay the temporal dimension of the feature to 1/2 of its original size, and accordingly, the channel size is increased to 1.5 times of its original size. In this way, by constructing four stages, each of which processes the dimensionality of the features, a different coarse fine-grained action representation can be obtained.

Next, the scale transformed feature token is fed into the Global block, which uses a multi-head self-attention mechanism to integrate global temporal context. Furthermore, its computation process can be described as follows: the input data $X={\left\{x_{i}\right\}}_{i=1}^{T}\epsilon R^{C\times T}$ go through eight head self-attention block, for each head $j\in \left\{1,\ldots ,8\right\}$, $x_{i}$ is projected using $W_{ji}^{q}$, $W_{ji}^{k}$, and $W_{ji}^{v}\epsilon R^{C/8\times C}$ to extract feature representations $Q_{ji}$, $K_{ji}$, and $V_{ji}$, referred to as query, key, and value. The outputs Q, K, and V are computed as $Q_{ji}=W_{ji}^{q}x_{i}$, $K_{ji}=W_{ji}^{k}x_{i}$, and $V_{ji}=W_{ji}^{v}x_{i}$. The output of the $j_{th}$ head self-attention is given by:

(1) $$A_{ji}=Softmax\left(Q_{ji}k_{ji}^{T}/\sqrt{\left.C/8\right.}\right)V_{ji}$$

Then, the combination of multi-head self-attention can be shown as:

(2) $$P_{i}=W_{i}^{O}Concat\left(A_{1i},\ldots ,A_{8i}\right)+x_{i}.$$

The output of multiple headers is concatted and then passed through a linear layer, where $W_{i}^{O}\epsilon R^{C\times C}$ denotes the weight of the linear layer. After the multi-head attention layer, the output feature size is the same as the input feature size. After that, we use two linear layers and a temporal convolution layer of kernel size 3 as the Local block to obtain the local temporal context. The first linear layer expands the feature dimension, then the convolution layer mixes the neighboring tokens to get local context, and finally, the last linear layer projects the feature dimension back.

Each Attenuation block contains L Global and Local blocks, and the final output from each Attenuation block is combined and fed into the Aggregation block.

**Aggregation Block.** After obtaining such multi-scale temporal features, we also need to aggregate the multi-scale features to have a unified video representation in order to facilitate the subsequent detection by the detection head, which requires our Aggregation block. The features obtained from each attenuation module are fed into the Aggregation block, as shown in Figure 4. We will first upsample the output of each Attenuation block $M_{n}$, $n\in \left\{1,\ldots ,N\right\}$, with different upsampling rates in different block; this operation can be formulated as:

(3) $$g_{n}\left(M_{n}\right)=UpSampling\left({\partial_{n}M}_{n}\right).$$

where $\partial_{n}\in R^{D_{o}\times \alpha^{n-1}C}$ denotes the weight of liner layer. Upsampling results in interpolation in the time dimension to the same time dimension as the input features.

Since the temporal and semantic information contained in the output features of the N Attenuation blocks varies greatly, in order to balance the temporal and semantic information between each output feature, each upsampled feature performs element-wise addition with the output of the Attenuation block N, which has also undergone the upsampling operation. This is because block N is the deepest layer of the network (N is taken to be 4 in our model), which contains the richest semantic information. This is given by:

(4) $$M_{n}^{\prime }=g_{n}\left(M_{n}\right)⊕g_{4}\left(M_{4}\right).$$

Finally, all the output of the Aggregation block will be concatenated to a final video representation:

(5) $$F_{HM}=Concat\left(M_{1}^{\prime },\ldots ,M_{4}^{\prime }\right).$$

where $F_{HM}$ represents the features obtained from the high-resolution stream after undergoing MRCA module processing.

### 3.4. Information Enhancement Module

Because the self-attention mechanism pays more attention to the correlation between positions but ignores the order and distance, we propose an additional convolution module as a complement to the self-attention mechanism: Information Enhancement module aims to aggregate long-range and short-range temporal context for temporal evaluation effectively and increase feature richness.

**Figure 5:**
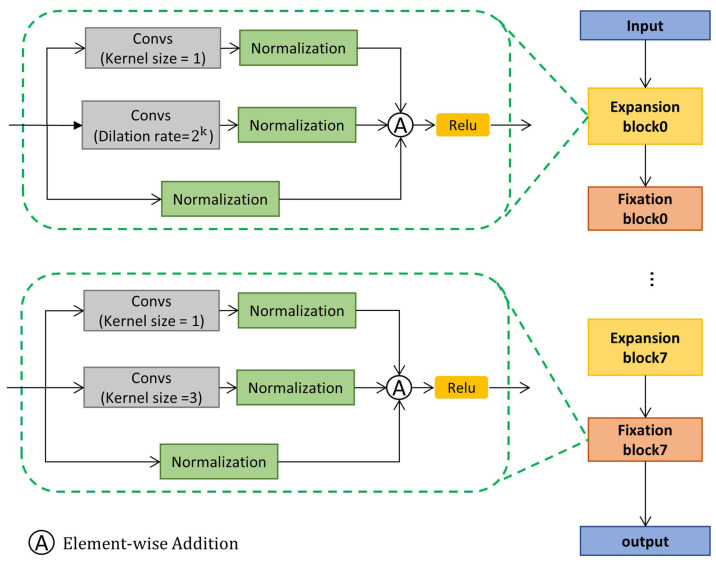
The architecture of the Information Enhancement module. First, temporal features are fed into the Expansion block with an increasing dilation to expand the receptive field. Then, the Fixation block with a fixed dilation smooths the features from the Expansion block.

As shown in Figure 5, the IE module can be divided into eight module groups, with two different blocks in each group: the Expansion block and Fixation block. The Expansion block consists of three paths. The first path is a short-range path including an ordinary convolution with a kernel size of 3, which aims to aggregate short-range temporal context. The second path is a long-range path, including a dilated convolution with a kernel size of 3 and a dilation rate of $2^{k}$, where k denotes the ordinal number of the current block. The role of this path is to aggregate long-range temporal context and expand the receptive field. The last path leaves the input features untouched in order to preserve the original information and alleviate network degradation during training. Finally, the final feature vector is obtained by fusing the output of the three paths. However, stacking a large number of dilated convolutions in the long-range path of the Expansion block can cause information loss, as not every position in the dilated convolution is involved in the computation, and therefore, information at some positions will be lost. In image processing, the solution to this problem is by stacking dilated convolutions with a jagged dilation rate so that the distribution of convolution kernels covers every position and there are no more omissions. The specific operation is to add a fixation block after each Expansion block, as shown in Figure 5. Compared with the Expansion block, the only change in the Fixation block is that the dilation rate of the expansion convolution in the long-range path is changed from $2^{k}$ to 3, so that the alternately connected Expansion block and Fixation block form the IE module, which can ensure that the receptive field grows rapidly and there is no information loss.

### 3.5. Norm and Localization

The input data, after being processed by the MRCA and IE modules, respectively, are then fed into the post-processing module and can be expressed by the following formula:

(6) $$F_{BNL}=\left[\left(F_{HM}+F_{LM}\right)/2\right]+F_{IE}.$$

where $F_{HM}$ and $F_{IE}$ represent features obtained from high-resolution streams processed by the MRCA and IE modules, respectively, and $F_{LM}$ denotes low-resolution streams processed by MRCA. The above features are fused to obtain the input $F_{BNL}$ for the post-processing module.

In the post-processing module, we first input features to the Norm module, using pre-defined anchors to generate segments ${\left\{U\right.}_{\varepsilon_{j}}{\}}_{j=1}^{J}$, where J is the total number of segments and $\varepsilon_{j}=\left\{t_{s,j},t_{e,j}\right\}$ represents the start moment and end moment of the j-th segment. We sample ϑ points (ϑ: alignment quantity) via interpolation and rescaling as described in Algorithm 1, and generate the segment feature $Y={\left\{y_{\varepsilon_{j}}\right\}}_{j=1}^{J}$.
**Algorithm 1** Interpolation and Rescaling in Norm&Location.**Input:** The input data ${\left\{x_{l}\right\}}_{l=1}^{L}$; ${\left\{U_{\varepsilon_{j}}\right\}}_{j=1}^{J}$, where *J* is the total number of processed data, $\varepsilon_{j}=\left\{t_{s,j},t_{e,j}\right\}$; alignment quantity ϑ;1:**for** each $U_{\varepsilon_{j}}$ **do**2:   List all $U_{\varepsilon_{j}}$ in chronological order;3:   Compute sampling interval $s=\left\lceil \left(t_{s,j}-t_{e,j}\right)/\vartheta \right\rceil$, interpolation quantity J=ϑs;4:   Sample *J* points based on linear interpolation using the two neighbors of each point $g=\left[t_{s}+\frac{k(t_{s,j}-t_{e,j})}{J}\;for\;k\;in\;range\left(J\right)\right]$;5:   $X_{in}=[\left(\left\lceil i\right\rceil -i\right)x_{\left\lfloor i\right\rfloor }+\left(i-\left\lfloor i\right\rfloor \right)x_{\left\lceil i\right\rceil }\;for\;i\;in\;g]$;6:   $y_{\varepsilon_{j}}=\left[mean\left(X_{in}\left[ks:\left[k+1\right]s\right]\right)\;for\;k\;in\;range\left(\vartheta \right)\right]$;7:**end for****Output:** *$Y={\left\{y_{\varepsilon_{j}}\right\}}_{j=1}^{J}$.*

For each segment $U_{\varepsilon }$, we calculate its Intersection-over-Union (IoU) with all ground-truth actions $G_{t}$, and denote the maximum IoU $\psi_{c}$ as the training target. Then, we set three fully connected layers(FC) for $U_{\varepsilon }$, while the last FC layer output two scores $p_{cls}$ and $p_{reg}$. They are trained to match $\psi_{c}$ using classification and regression losses, respectively.

### 3.6. Training and Inference

**Training.** We train MCMNET by using the classification loss $L_{c}$ and the localization loss $L_{r}$:

(7) $$L=L_{r}+L_{c}.$$

The loss $L_{r}$ is used to determine the confidence scores of segments. The loss $L_{c}$ classifies each feature segment according to its position relative to the action. During model training, the training set in the dataset is processed into two resolutions and then input into the model; the two resolution streams are, respectively, passed through the MRCA module to get the spatio-temporal features with higher-level semantic information, after which the high-resolution streams are passed through the IE module to get the features with long-term and short-term temporal information, and then the two features are united to the same resolution and then fused to get the final video features $F_{BNL}$. Inputting $F_{BNL}$ into the Norm&Localization module yields two scores, $p_{cls}$ and $p_{reg}$. With these two parameters, the first part of the loss function $l_{r}$ can be constructed. The localization loss $L_{r}$ is defined as follows:

(8) $$L_{r}=L_{wce}\left(p_{cls},G_{cls}\right)+\omega_{1}\cdot L_{mse}\left(p_{reg},\psi_{c}\right).$$

where $L_{wce}$ is the weighted cross-entropy loss function used to calculate the loss of $p_{cls}$ and $G_{cls}$ and $G_{cls}$ is the confidence score obtained by binarizing the IoU map with a threshold 0.5, which is calculated by proposals and ground truth. Furthermore, $L_{mse}$ is the square error loss. The weight is computed to balance the positive and negative training samples and we set the weighting factor $\omega_{1}$ to 10.

Meanwhile, we input Fbnl directly into FC layers to get the starting and ending probabilities ($p_{s}$, $p_{e}$) and corresponding training targets for each feature segment ($d_{ss}$,$d_{se}$). With these two parameters, it is possible to derive the classification loss function $L_{c}$:

(9) $$L_{c}=L_{wce}\left(p_{s},d_{ss}\right)+L_{wce}\left(p_{e},d_{se}\right).$$

Finally, we use the weighted cross-entropy loss $L_{wce}$ to calculate the difference between the prediction and the target.

**Inference.** During inference, the previous method for data processing is the same as in training, and the video data are passed through the trained model to get the final video feature $F_{BNL}$. After feeding $F_{BNL}$ into the Norm&Localization module, MCMNET outputs the classification and regression scores for each segment $U_{\varepsilon }$. Among J segments, we construct:

(10) $$\Lambda ={\left\{\lambda_{j}=\left({\overset{-}{t}}_{s,j},{\overset{-}{t}}_{e,j},{\overset{-}{C}}_{j},P_{j}\right)\right\}}_{j=1}^{J}.$$

where $\left({\overset{-}{t}}_{s,j},{\overset{-}{t}}_{e,j}\right)$ indicate the beginning and end moment of the predicted action, ${\overset{-}{C}}_{j}$ denotes the predicted action class, and $P_{j}$ denotes the confidence score of the prediction. $P_{j}$ is obtained by $P_{j}=p_{cls}^{\alpha }\cdot p_{reg}^{1-\alpha }$, where α is obtained by searching in each setup after optimal value in the experiment. By comparing the predicted segment with the ground-truth, it is possible to obtain the mAP at different tIoUs (temporal Intersection over Union).

## 4. Experiments

### 4.1. Datasets and Metrics

We perform extensive experiments on the datasets of ActivitiesNet-v1.3, Charades, and TSU to demonstrate the effectiveness of our MCMNET. For comparison with existing models, our work follows the standard evaluation scheme and uses the mAP with intersection over union(IoU) thresholds as the evaluation metric.

**ActivityNet-v1.3** [58] is a large-scale dataset containing 10,024 training videos, 4926 validation videos, and 5044 test videos belonging to 200 activities covering sports, household, and working actions. ActivitiesNet-v1.3 only contains 1.5 occurrences per video on average, and most videos contain a single action category with 36% background on average. We report the mAP with IoU thresholds [0.5, 0.75, 0.95] on ActivitiyNet-v1.3.

**Charades** [59] is a densely labeled dataset with 9848 videos of 157 daily indoor actions, separated into 7.9 k training and 1.8 k validation clips. Each video may include multiple overlapping activities annotated with frame-level labels. This is in contrast to ActivityNet, which only has one action per time-step. The average length of a video is 30 s. We evaluate the per-frame mAP on these densely labeled datasets following [60,61].

**TSU** [62] (Toyota Smarthome Untrimmed) is also a densely labeled dataset that contains 536 videos with an average duration of 21 min. Besides, TSU contains some very similar actions, such as eating food and drinking a drink, and some actions with high temporal variance, such as putting on glasses in 5 s, reading for 10 min, or some subtle actions such as stirring coffee. As a result, TSU has longer action durations and more complex temporal relationships than other datasets. We evaluate the per-frame mAP on TSU as Charades.

**MultiTHUMOS** [61] is an extended version of THUMOS’14 [63] dataset, which contains dense, multilabel, frame-level action annotations for 30 h across 400 videos in the THUMOS’14 action detection dataset. It consists of 38,690 annotations of 65 action classes, with an average of 1.5 labels per frame and 10.5 action classes per video. This is in contrast to other activity detection datasets, such as ActivityNet and HACS(Human Action Clips and Segments) [64], which only have one activity per time-step.

### 4.2. Implementation Details

We use pre-extracted features for these three datasets. For ActivityNet-v1.3 and Charades, we used the pre-trained dual-stream network of [65] to extract video feature. For TSU, we use the officially available RGB I3D feacure. In the proposed network, the number of Attenuation blocks and Aggregation blocks is set to B = 4 and the number of Expansion blocks and Fixation blocks is set to N = 8. The number of attention heads for Global block is set to 8. Finally, we implemented and compiled our framework by using PyTorch 1.9, Python 3.7, and CUDA 11.6. For Charades and TSU training, we set the learning rate, batch size, and epoch to 0.0003, 24, and 10, respectively. In ActivityNet-1.3 training, the above parameters are set to 0.00003, 30, and 6, respectively. The learning rate will drop 10-fold every epoch.

### 4.3. Comparison with State-of-the-Arts Methods

In this subsection, we compare MCMNET with the state-of-the-art action detection method on ActivityNet-v1.3, Charades, MultiTHUMOS, and TSU in Table 1 and Table 2.

In ActivityNet-v1.3, MCMNET is less advantageous for this dataset, as it contains fewer action instances per video and the temporal relationships are relatively simple, whereas the focus of MCMNET is on modeling multi-scale temporal contextual information. Furthermore, by comparing with the mainstream temporal action location methods, it is found that MCMNET performs significantly better than other methods when the IoU requires medium accuracy. When we focus on densely labeled datasets, we find that MCMNET performs reasonably well compared to other methods for these videos with more complex temporal relationships. Benefiting from MCMNET’s excellent multi-scale temporal information aggregation capability, it performs outstandingly on Charades and MultiTHUMOS. Although MCMNET did not achieve the best performance on the TSU dataset, it still reached the state-of-the-art level. This is probably due to the fact that our model has a large number of parameters, while the TSU data volume is much smaller than that of Charades, and there are many longer video and action instances in the TSU. Therefore, we also need to pay more attention to modeling temporal information over long distances. At the same time, we compared the computational consumption required by several models, and from the results, we can see that our model requires much less computational resources than the model with pure self-attention mechanism (MLAD), but the computational consumption will still be higher than that of the model with pure CNN models (PDAN, TGM) because our model needs to take care of both the long duration actions and the short duration actions. In the future, we hope to simplify the model as much as possible and reduce the number of parameters so that we can achieve a better balance between effectiveness and efficiency.

### 4.4. Ablation Study

In this subsection, we validate the effectiveness of different components of MCMNET and evaluate the effects of various hyper-parameters.

**Effectiveness of MRCA.** The MRCA module can be divided into two components: Attenuation block and Aggregation block. The Attenuation block can be further divided into three submodules: Reduction block, Global block, and Local block. We ablate the four submodules and study their impact on the final performance. Each submodule is individually enabled and disabled. We conduct ablation experiments on ActivityNet-v1.3, Charades, and TSU, respectively; the results are shown in Table 3 and Table 4. It can be seen that overall, the Attenuation block has a significant improvement in the performance on all three datasets, which proves the effectiveness of the module for aggregating local and global temporal contexts. Moreover, the Global block has a more obvious improvement in the two densely labeled datasets, suggesting that self-attention is well suited for handling data with complex temporal relationships.

**Effectiveness of IE.** The IE module contains Expansion block and Fixation block, where the Expansion block is used to expand the receptive field and to aggregate long-range temporal context, the Fixation block is used to prevent grid artifact caused by the rapid expansion of receptive field by the Expansion block. The results in Table 5 and Table 6 show that when only Expansion block is present, there is a drop in accuracy for all three datasets. This is due to the fact that a large number of stacked dilated convolution layers can cause certain positions in the feature maps to be skipped and fail to participate in the computation. However, when both the Expansion block and the Fixation block are present, the results are much improved.

**Choice of attenuation factor.** In the MACA module, each Attenuation block reduces the time dimension of the input feature to build multi-scale temporal context information. Furthermore, the attenuation factor greatly affects the final feature quality. Therefore, we conducted ablation experiments on the high-resolution stream and the low-resolution attenuation factor, respectively, and the results are shown in Table 7. The results show that the best results can be achieved when the attenuation factor of the high-resolution stream is 2 and that of the low-resolution stream is 1.5. Besides, it can be seen from the results that the effect of the attenuation factor is greater in the high resolution than in the low resolution.

**Choice of the number for IE block.** In the IE module, we stack multiple Expansion blocks and Fixation blocks to aggregate long-range and short-range temporal context. We also conducted ablation experiments to determine the optimal number of blocks to use, and the results are shown in Table 8. The results show that as the number of blocks increases from 1 to 7, the performance gradually improves, but when the number of blocks is higher, the accuracy begins to decrease. This is because the kernel size of dilated convolution in the Expansion block is $2^{k}$, and as the number of layers increases, the receptive field quickly expands. When it exceeds a certain limit, information loss occurs, which leads to a decrease in accuracy. Therefore, we set the final number of blocks to 7.

**Efficiency Analysis.** In this part, we report the effect of each module of MCMNET on the inference time and GFLOPs on ActivityNet-v1.3. Using 2000 proposals as input to the model and processing the video using NVIDIA RTX 3080ti for about 20 min, the results are shown in Table 9. The overall time required for the MRCA module is greater than that for the IE module. This is mainly because the multi-head self-attention in the MRCA module processes features at different time resolutions, which requires a large amount of computation. In addition, the Expansion block in the IE module requires much more time than the Fixation block. This is because in the later blocks of the IE module, the dilated convolution kernel size in the Expansion block is already very large, and the convolution operation at this stage also requires a long time. From the computational consumption results, it can be seen that overall, the computational resources required by the IE module are higher than those of the MRCA, which is due to the fact that the CNN network requires deeper network layers in order to achieve higher perceptive field. Therefore, we also need to work on model simplification in the future so that higher efficiency can be achieved.

### 4.5. Visualization

**Figure 6:**
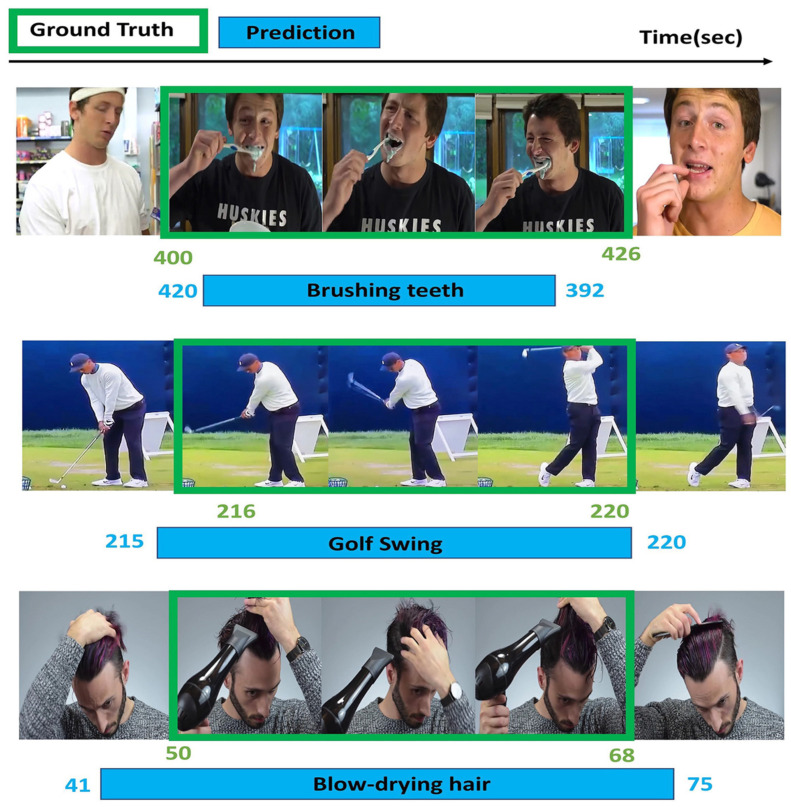
Qualitative results. We show the qualitative results on TSU (**top**), ActivityNet-v1.3 (**middle**, and Charades (**bottom**).

We show some qualitative detection results in Figure 6 on TSU (top), ActivityNet-v1.3 (middle) and Charades (bottom). The results showed that the detection performance was very good for some short-duration actions, such as a golf swing. However, the performance still needs to be improved for some longer duration actions, such as brushing teeth and blow drying hair.

**Figure 7:**
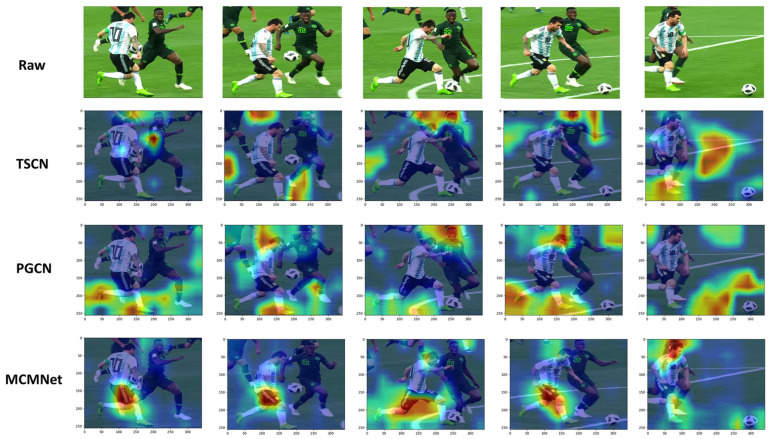
Visualization of activation maps with GradCAM. Activation maps generated by TSCN, PGCN, and our MCMNET for the action “playing soccer”. Compared to TSCN and PGCN, it can be noted that MCMNET can focus more precisely on the subject of the action rather than on the irrelevant background.

We also used GradCAM [73] to visualize the class activation map of three models, TSCN, PGCN, and our MCMNET, as shown in Figure 7. From the visualization results, it can be seen that for kicking a football, MCMNET is more precise in locating the key parts compared to the other two models. It focuses mainly on a few key movement parts and rejects the background unrelated to the movement.

## 5. Conclusions

In this paper, we have proposed a multi-scale context modeling network. First, we extract the feature sequence from the video using a pre-trained model and splitting it into a high-resolution stream and a low-resolution stream. The two streams are then fed into the MRCA module to obtain local and global temporal contexts. The high-resolution streams are additionally fed into the IE module for modeling long-range and short-range temporal relationships. Finally, the three output features are fused and passed through the Norm&location module to regularize the data, which is then input into the classifier to obtain the final score. Extensive experiments conducted on three challenging action detection benchmarks demonstrate that our MCMNET achieves outstanding temporal localization performance.

However, there are still some shortcomings in our approach, the main one being that the overall simplicity of the model is neglected in order to achieve better results. On the other hand, since our model operates on pre-extracted features, the whole model cannot be trained end-to-end with raw video data as input. This also leads to the fact that we cannot explore in detail the effect of spatial features of video data on the detection effect. In the future, we will explore in more detail the combination of modules which can take advantage of the strengths of each module while maintaining the simplicity of the model structure. Furthermore, moving closer to an end-to-end training model, it is possible to better model both temporal and spatial features and build a more robust model.

## Figures and Tables

**Table 1:** Action detection results on the validation set of ActivityNet-1.3 measured by mAP (%) at different tIoU thresholds and the average mAP.

Method	0.5	0.75	0.95	Average
SCC [66]	40.00	17.90	4.70	21.70
CDC [3]	45.30	26.00	0.20	23.80
BSN [29]	46.45	29.96	8.02	30.03
PGCN [6]	48.26	33.16	3.27	31.11
BMN [35]	50.07	34.78	8.29	33.85
TSCN [67]	35.30	21.40	5.30	21.70
G-TAD [7]	50.36	34.60	9.02	34.09
E2E-TAD [68]	50.47	35.99	**10.83**	35.10
Actionformer [54]	**53.50**	**36.20**	8.2	**35.6**
TadTR [53]	49.12	32.58	8.63	32.30
MCMNET (ours)	46.70	34.90	6.38	32.83

**Table 2:** Action detection results on validation set of Charades, TSU, and MultiTHUMOS. Note that the evaluation for the methods is based on per-frame mAP (%) using only RGB videos.

Method	Charades	TSU	MultiTHUMOS	GFLOPs
R-C3D [4]	12.7	8.7	-	-
PDAN [67]	23.7	32.7	40.2	3.2
TGM [69]	20.6	26.7	37.2	1.2
MS-TCT [70]	25.4	**33.7**	**43.1**	6.6
TTM [71]	**28.8**	-	-	0.8
MLAD [72]	18.4	-	42.2	44.8
MCMNet (ours)	27.3	33.1	43.0	24.7

**Table 3:** **Ablating MRCA Components on ActivityNet-v1.3.** We disable Reduction block/Global block/Local block/Aggregation block on ActivityNet-v1.3.

Reduction Block	Global Block	Local Block	Aggregation Block	0.5	0.75	0.95	Avg.
×	×	×	×	40.88	26.93	2.57	25.71
✓	×	×	×	41.92	27.35	2.92	27.96
✓	✓	×	×	42.61	30.78	4.03	28.06
✓	✓	✓	×	44.02	32.18	5.65	30.73
✓	✓	✓	✓	**46.70**	**34.90**	**6.38**	**32.83**

**Table 4:** **Ablating MRCA Components on Charades and TSU.** The evaluation is based on per-frame mAP on the Charades and TSU datasets.

Reduction Block	Global Block	Local Block	Aggregation Block	Charades	TSU
×	×	×	×	25.9	31.4
✓	×	×	×	26.3	32.0
✓	✓	×	×	26.8	32.3
✓	✓	✓	×	27.1	32.9
✓	✓	✓	✓	**27.3**	**33.1**

**Table 5:** **Ablating IE Components on Activity-v1.3.** We verify the usefulness of the Expansion block and Fixation block on ActivityNet-v1.3.

Expasion Block	Fixation Block	0.5	0.75	0.95	Avg.
×	×	41.02	28.51	5.18	30.38
×	✓	43.97	29.78	5.69	31.14
✓	×	41.48	31.22	5.10	30.81
✓	✓	**46.70**	**34.90**	**6.38**	**32.83**

**Table 6:** **Ablating IE Components on Charades and TSU.** We also tested the effect of the IE module on the Charades and TSU dataset.

Expasion Block	Fixation Block	Charades	TSU
×	×	26.1	32.0
×	✓	26.3	32.7
✓	×	25.9	32.1
✓	✓	**27.3**	**33.1**

**Table 7:** **The effect of different attenuation factor** on Charades and TSU.

$\alpha_{1}$	$\alpha_{2}$	Charades	TSU
1	1	26.2	32.1
1	1.5	26.7	32.7
2	1	27.0	33.0
2	1.5	**27.3**	**33.1**
2	2	27.2	32.9
4	2	26.5	32.6

**Table 8:** **The effect of different block numbers of IE module** on Charades and TSU.

Block Number N	Charades	TSU
1	26.6	32.1
3	26.8	32.6
5	27.1	32.9
7	**27.3**	**33.1**
9	27.2	32.8
11	27.0	32.5

**Table 9:** The inference time and GFLOPs of each module in MCMNET on the ActivityNet-v1.3. 2000 candidate proposals were utilized as input to MCMNET, and an Nvidia 3080Ti graphic card was employed to process a video for about 20 min.

MRCA	IE (Expansion Block)	IE (Fixation Block)	Tcost (s)	GFLOPs
✓	×	×	0.133	10.2
✓	✓	×	0.171	17.5
✓	x	✓	0.290	17.4
✓	✓	✓	0.312	24.7

## Data Availability

The datasets generated during and analysed during the current study and source code are all available from the corresponding author on reasonable request.
